# ∗-fuzzy measure model for COVID-19 disease

**DOI:** 10.1186/s13662-021-03363-3

**Published:** 2021-04-12

**Authors:** Abbas Ghaffari, Reza Saadati

**Affiliations:** 1 Department of Mathematics, Science and Research Branch, Islamic Azad University, Tehran, Iran; 2 School of Mathematics, Iran University of Science and Technology, Narmak, Tehran, Iran

**Keywords:** 46S40, Fuzzy measure, Mathematical model, COVID-19 disease, Fuzzy integration, t-norm

## Abstract

We introduce a mathematical model, namely, ∗-fuzzy measure model for COVID-19 disease and consider some properties of ∗-fuzzy measure such as Lebesque–Radon–Nikodym theorem.

## Introduction

In this paper, we consider a new mathematical model named ∗-fuzzy measure model. The idea for this mathematical model is taken from the state of our world today, which is involved in the outbreak of COVID-19. Let us assume that the universal set in our study, ϒ, is the human community, and the family of all subsets of ϒ is the *σ*-algebra that we denote by $\mathcal{M}$. Now, if we describe the destructive effect of the disease on each subset of ϒ with a fuzzy number from $(0,1]$, then, due to the lack of knowledge and transparency about this disease, the study population becomes larger and (dealing with this problem) is more complicated. Therefore, as the subsets grow, the fuzzy number assigned to it decreases, and the only ideal subset is the empty set that at every time, $t_{\alpha }$, has fuzzy number 1. Now, if we consider two fuzzy numbers related to two sets *ω* and *κ*, according to above, we have

$$\begin{aligned} \text{fuzzy number } (\omega \cup \kappa )\leq \min \lbrace \text{ fuzzy number } \omega , \text{fuzzy number } \kappa \rbrace , \end{aligned}$$

then, in the best situation, if $\omega \cap \kappa =\emptyset $ and ∗ is a binary operation on the unit interval $(0,1]$ that is smaller than the minimum (Archimedean property $\delta \ast \delta <\delta $) such that

$$\begin{aligned} \text{fuzzy number } (\omega \cup \kappa )= ( \text{fuzzy number } \omega ) \ast (\text{fuzzy number } \kappa ). \end{aligned}$$

Using the described model, we can define the following dynamical function:

$$\begin{aligned} \mu ^{\blacktriangle } : \mathcal{M}\times (0, +\infty )\longrightarrow [0,1] \end{aligned}$$

such that

(i) $\mu ^{\blacktriangle }(\emptyset ,t_{\alpha })=1$ for every $t_{\alpha }\in (0,+\infty )$ where $t_{\alpha }$ is time parameter;
(ii) If $\omega \cap \kappa =\emptyset $ then $\mu ^{\blacktriangle }(\omega \cup \kappa ,t_{\alpha })=\mu ^{ \blacktriangle }(\omega ,t_{\alpha })\ast \mu ^{\blacktriangle }( \kappa ,t_{\alpha })$;
(iii) If $\omega _{j}\cap \omega _{l}=\emptyset $ whenever $j\neq l$ then $\mu ^{\blacktriangle }(\bigcup_{j=1}^{n} \omega _{j},t_{ \alpha })=\ast _{j=1}^{n} \mu ^{\blacktriangle }(\omega _{j},t_{\alpha })$,where $\mu ^{\blacktriangle }$ is named ∗-fuzzy measure.

Note that for $\kappa \in \mathcal{M }$ and when $t_{\alpha }$ tends to 0 we confront the worst possible situation while we are interested in the set *κ* reaching the best possible (eliminating disease) situation by planning and managing over time. Then,

1.1 $$\begin{aligned} \lim_{t_{\alpha }\rightarrow 0}\mu ^{\blacktriangle }( \kappa ,t_{ \alpha })=0 , \qquad \lim_{t_{\alpha }\rightarrow +\infty }\mu ^{\blacktriangle }(\kappa ,t_{\alpha })=1. \end{aligned}$$

The dynamic fuzzy measure, integration theory, and norm-based ∗-fuzzy $(L^{+})^{p}$ spaces were introduced in [4]. Now we continue the previous discussion and investigate the dual of $(L^{+})^{p}$ ∗-fuzzy normed spaces. For new results in other situations, we can suggest some sources, e.g., [7–9, 12].

## ∗-Fuzzy measure

Let $\Upsilon \neq\emptyset $ and $\mathcal{M}$ be a *σ*-algebra on ϒ. Also we put $I=[0,1]$ and $J=[0,+\infty )$.

### Definition 2.1

([5, 13])

We define a continuous triangular norm (abbreviated *ct*-norm) as a continuous binary operation,

$$\begin{aligned} \ast : [0,1]\times [0,1]\longrightarrow [0,1], \end{aligned}$$

such that

(i) $\theta \ast \theta ^{\prime } = \theta ^{\prime }\ast \theta $, for all $\theta ,\theta ^{\prime }\in I$,
(ii) $\theta \ast (\theta ^{\prime }\ast \theta '')= (\theta \ast \theta ^{ \prime })\ast \theta '' $, for all $\theta ,\theta ^{\prime },\theta ''\in I$,
(iii) $\theta \ast 1=\theta $, for all $\theta \in I$,
(iv) If $\theta _{1}< \theta _{2}$ and $\theta ^{\prime }_{1}< \theta ^{\prime }_{2}$ then $\theta _{1}\ast \theta ^{\prime }_{1}< \theta _{2}\ast \theta ^{ \prime }_{2}$, for all $\theta _{1}\theta _{2},\theta ^{\prime }_{1}, \theta ^{\prime }_{2} \in I$.

Now, we consider some examples of t-norms:

1. $\theta \ast \theta ^{\prime }=\max \lbrace \theta +\theta ^{\prime }-1,0 \rbrace $, where we put $\ast =\ast _{L}$ – the Lukasiewicz t-norm;
2. $\theta \ast \theta ^{\prime }= \theta \cdot \theta ^{\prime }$, where we put $\ast =\ast _{P}$ – the product t-norm;
3. $\theta \ast \theta ^{\prime }= \big\{ \scriptsize{ \begin{array}{l@{\quad}l} 0, & \text{if } \theta =\theta ^{\prime }=0, \\ \frac{1}{\frac{1}{\theta }+\frac{1}{\theta ^{\prime }}-1}, & \theta \text{ or } \theta ^{\prime }\neq 0, \end{array} } $ where we put $\ast =\ast _{H}$ – the Hamacher t-norm;
4. $\theta \ast \theta ^{\prime }=\min \lbrace \theta ,\theta ^{\prime } \rbrace $, where we put $\ast =\ast _{M}$ – the minimum t-norm.

Note that, the *ct*-norm is said to be Archimedean (abbreviated $cat$-norm) when $\delta \ast \delta < \delta $ for every $\delta \in I^{o}$ (interior of *I*). For example, $\ast _{P}$, $\ast _{H}$, and $\ast _{L}$ are $cat$-norms, but $\ast _{M}$ is not. Throughout this paper we use a $cat$-norm.

### Definition 2.2

Let ϒ be a nonempty set, $\mathcal{M}$ be a *σ*-algebra, and let ∗ be a $cat$-norm. The fuzzy set $\mu ^{\blacktriangle }$,

$$\begin{aligned} \mu ^{\blacktriangle } : \mathcal{M}\times J^{o}\longrightarrow I, \end{aligned}$$

is said to be a ∗-fuzzy measure (abbreviated ∗-FM) whenever

(i) $\mu ^{\blacktriangle }(\emptyset ,t_{\alpha })=1$, for all $t_{\alpha }\in J^{o}$;
(ii) $\mu ^{\blacktriangle } (\bigcup_{j=1}^{ +\infty } \omega _{j},t_{\alpha } )=\ast _{j=1}^{ +\infty } \mu ^{\blacktriangle }(\omega _{j},t_{\alpha })$, for all $t_{\alpha }\in J^{o}$ in which $\omega _{j}\in \mathcal{M}$, $j=1,2,\dots $, and $\omega _{j}\cap \omega _{l}=\emptyset j\neq l$.

Note that, a ∗-FM $\mu ^{\blacktriangle }$ is finitely additive if

$$\begin{aligned} \mu ^{\blacktriangle } \Biggl(\bigcup_{j=1}^{n} \omega _{j},t_{ \alpha } \Biggr)=\ast _{j=1}^{n} \mu ^{\blacktriangle }(\omega _{j},t_{ \alpha }), \quad \text{for all } t_{\alpha } \in J^{o} , \end{aligned}$$

in which $\omega _{j}\in \mathcal{M}$, $j=1,2,\dots ,n$, and $\omega _{j}\cap \omega _{l}=\emptyset $, $j\neq l$.

The quadruple $(\Upsilon ,\mathcal{M},\mu ^{\blacktriangle },\ast )$ is said to be a ∗-FM space (abbreviated ∗-FMS). Now, we give some examples.

### Example 2.3

Suppose $(\Upsilon ,\mathcal{M},\mu )$ is a measure space and $\ast =\ast _{P}$. We set,

$$\begin{aligned}& \mu ^{\blacktriangle }: \mathcal{M}\times J^{o}\longrightarrow I, \\& \mu ^{\blacktriangle }(\kappa ,t_{\alpha })=e^{ \frac{-\mu (\kappa )}{t_{\alpha }}},\quad \text{for all } t_{\alpha } \in I^{o}. \end{aligned}$$

Thus $\mu ^{\blacktriangle }$ is a ∗-FM.

### Example 2.4

Consider the measure space $(\Upsilon ,\mathcal{M},\mu )$ and $\ast =\ast _{P}$. We set,

$$\begin{aligned}& \mu ^{\blacktriangle }: \mathcal{M}\times J^{o}\longrightarrow I, \\& \mu ^{\blacktriangle }(\omega ,t_{\alpha })= \textstyle\begin{cases} e^{\frac{-\mu (\omega )}{\sin t_{\alpha }}}, & t_{\alpha }\in (2m \pi ,(2m+1)\pi ), \\ e^{\frac{\mu (\omega )}{\sin t_{\alpha }}}, & t_{\alpha }\in ((2m+1) \pi ,(2m+2)\pi ), \\ e^{\frac{-\mu (\omega )}{1+\sin t_{\alpha }}}, & t_{\alpha } =2m\pi \text{or} t_{\alpha }=(2m+1)\pi , \end{cases}\displaystyle \end{aligned}$$

thus $\mu ^{\blacktriangle }$ is a ∗-FM.

### Example 2.5

Consider the measure space $(\Upsilon ,\mathcal{M},\mu )$ and $\ast =\ast _{H}$. We set,

$$\begin{aligned}& \mu ^{\blacktriangle }: \mathcal{M}\times J^{o}\longrightarrow I, \\& \mu ^{\blacktriangle }(\omega ,t_{\alpha })= \frac{t_{\alpha }}{t_{\alpha }+\mu (\omega )}, \quad \text{for all } t_{ \alpha }\in J^{o}. \end{aligned}$$

Thus $\mu ^{\blacktriangle }$ is a ∗-FM.

### Remark

In Example 2.4 the ∗-FM $\mu ^{\blacktriangle }$ is not monotone or continuous in the second place, but in Examples 2.3 and 2.5 ∗-FM $\mu ^{\blacktriangle }$ is monotone and continuous. Thus, we assume that

$$\begin{aligned} \mu ^{\blacktriangle }(\omega ,.):J^{o}\longrightarrow I, \end{aligned}$$

is increasing and continuous. Also, we let every ∗-FM satisfy (1.1).

### Theorem 2.6

([4])

*Consider the* ∗-*FMS* $(\Upsilon ,\mathcal{M},\mu ^{\blacktriangle },\ast )$.

(i) *If* $\omega ,\kappa \in \mathcal{M}$ *and* $\omega \subseteq \kappa $ *then*
  $$\begin{aligned} \mu ^{\ast }(\omega ,t_{\alpha })\geq \mu ^{\blacktriangle }( \kappa ,t_{ \alpha }),\quad \textit{for all } t_{\alpha }\in (0, + \infty ); \end{aligned}$$
(ii) *If* $\omega _{j}\in \mathcal{M}$ *and* $\omega =\bigcup_{j=1}^{+\infty }\omega _{j}$ *then*
  $$\begin{aligned} \mu ^{\blacktriangle } \Biggl(\bigcup_{j=1}^{ +\infty } \omega _{j} \Biggr)\geq \ast _{j=1}^{ +\infty }\mu ^{\blacktriangle }(\omega _{j},t_{\alpha }),\quad {\textit{for all }} t_{\alpha }\in (0,+\infty ); \end{aligned}$$
(iii) (*continuity from below*) *Suppose* $\omega _{j}\in \mathcal{M}$ *and* $\omega _{j}\uparrow \omega =\bigcup_{j=1}^{ +\infty }\omega _{j}$. *Then*,
  $$\begin{aligned} \mu ^{\blacktriangle } \Biggl(\bigcup_{j=1}^{ +\infty } \omega _{j},t_{\alpha } \Biggr)=\lim_{j\to +\infty } \mu ^{\blacktriangle }(\omega _{j},t_{\alpha }), \quad {\textit{for all }} t_{\alpha }\in (0,+\infty ); \end{aligned}$$
(iv) (*continuity from above*) *Suppose* $\omega _{j}\in \mathcal{M}$ *and* $\omega _{j}\downarrow \omega =\bigcap_{j=1}^{ +\infty }\omega _{j}$. *Then*,
  $$\begin{aligned} \mu ^{\blacktriangle } \Biggl(\bigcap_{j=1}^{ +\infty } \omega _{j},t_{\alpha } \Biggr)=\lim_{j\to {+ \infty }} \mu ^{\blacktriangle }(\omega _{j},t_{\alpha }), { \quad \textit{for all }} t_{\alpha }\in (0,+\infty ). \end{aligned}$$

### Definition 2.7

Suppose that $\mu ^{\blacktriangle }$ is a ∗-FM. We say $\mu ^{\blacktriangle }$ is bounded if $\mu ^{\blacktriangle }(\Upsilon ,t_{\alpha })>0$ for each $t_{\alpha }\in J^{o}$ and $\mu ^{\blacktriangle }$ is *σ*-bounded if there exist sequence $\lbrace \omega _{j}\rbrace \subseteq \mathcal{M}$ for $j\in \mathbb{N}$ such that $\mu ^{\blacktriangle }(\omega _{j},t_{\alpha })>0$ for each $j\in \mathbb{N}$ and $\Upsilon =\bigcup_{j=1}^{+\infty }\omega _{j}$. Also we say $\mu ^{\blacktriangle }$ is ∗-fuzzy pseudobounded if for every $\omega \in \mathcal{M}$ with $\mu ^{\blacktriangle }(\omega ,t_{\alpha })=1$, there exists $\kappa \in \mathcal{M}$ such that $\kappa \subset \omega $ and $0< \mu ^{\blacktriangle }(\kappa ,t_{\alpha })<1$. If $\mu ^{\blacktriangle }$ is a bounded ∗-FM, then $(\Upsilon ,\mathcal{M},\mu ^{\blacktriangle },\ast )$ is said to be a bounded ∗-FMS, similarly, if $\mu ^{\blacktriangle }$ is a *σ*-bounded ∗-FM, then $(\Upsilon ,\mathcal{M},\mu ^{\blacktriangle },\ast )$ is said to be a *σ*-bounded ∗-FM.

## Measurable functions

The concept of ∗-fuzzy normed spaces (∗-fNLS) was defined in [2, 6, 14].

### Definition 3.1

Let ϒ be a vector space, ∗ be a *ct*-norm, and let the fuzzy set $N^{\blacktriangle }$ on $\Upsilon \times (0,+\infty )$ satisfy the following conditions for all $\xi ,\zeta \in \Upsilon $ and $t_{\alpha },t_{\beta } \in (0,+\infty )$:

(i) $N^{\blacktriangle }(\xi ,t_{\alpha }) >0$;
(ii) $N^{\blacktriangle }(\xi ,t_{\alpha })=1$ if and only if $\xi =0$;
(iii) $N^{\blacktriangle }(c\xi ,t_{\alpha })= N^{\blacktriangle } (\xi , \frac{t_{\alpha }}{c} )$, for every $c>0$;
(iv) $N^{\blacktriangle }(\xi ,t_{\alpha })\ast N^{\blacktriangle }(\zeta ,t_{ \beta }) \leq N^{\blacktriangle }(\xi +\zeta ,t_{\alpha }+t_{\beta })$;
(v) $N^{\blacktriangle }(\xi ,.) : (0,+\infty ) \rightarrow (0,1]$ is continuous;
(vi) $\lim_{t_{\alpha } \rightarrow +\infty } N^{\blacktriangle }( \xi ,t_{\alpha })=1$ and $\lim_{t_{\alpha } \rightarrow 0} N^{\blacktriangle }(\xi ,t_{ \alpha })=0$.

Then, $N^{\blacktriangle }$ is called a ∗-fuzzy norm on ϒ and $(\Upsilon ,N^{\blacktriangle },\ast )$ is called ∗-fNLS.

### Lemma 3.2

([3, 11])

*Let* $(\mathbb{R}, N^{\blacktriangle }, \ast )$ *be* ∗-*fNLS*. *Then* $\omega \subseteq \mathbb{R}$ *is fuzzy bounded if and only if* $\omega \subseteq \mathbb{R}$ *is bounded in* $(\mathbb{R},\vert \cdot \vert )$.

### Definition 3.3

Consider the measure spaces $(\Upsilon ,\mathcal{M})$ and $(Z,\mathcal{N})$. The map $\xi :\Upsilon \rightarrow Z$ is said to be ($\mathcal{M}$–$\mathcal{N}$)-measurable if $\xi ^{-1}(\kappa )\in \mathcal{M}$ for every $\kappa \in \mathcal{N}$.

We denote by $\mathcal{B}_{\Upsilon }$ the Borel *σ*-algebra on ϒ.

### Theorem 3.4

([1, 10])

*If* $\xi :\Upsilon \rightarrow \mathbb{R}$ *is a measurable function*, *thus there is a sequence* $\{\psi _{n}\}$ *of simple functions such that* $0\leq \psi _{1}\leq \psi _{2}\leq \cdots \leq \xi $ *pointwise*.

## ∗-Fuzzy integration

Now, we study ∗-fuzzy integration and its properties.

### Definition 4.1

Suppose that $(\Upsilon ,\mathcal{M},\mu ^{\blacktriangle },\ast )$ is a ∗-FMS. We put

$$\begin{aligned} L_{+}= \bigl\lbrace \xi :\Upsilon \rightarrow \mathbb{R}\mid \xi \text{ is }(\mathcal{M}, \mathcal{B}_{\mathbb{R}})\text{-measurable} \bigr\rbrace . \end{aligned}$$

We consider $\psi (\upsilon )=\sum_{j=1}^{n}b_{j}\chi _{\kappa _{j}}( \upsilon )$, for every $b_{j}> 0$ where $\kappa _{j}\in \mathcal{M}$ for $j\in \mathbb{N}$, we define the ∗-fuzzy integral of *ψ* as

$$\begin{aligned} \int _{\Upsilon }\psi (\upsilon )\,d\mu ^{\blacktriangle }(\upsilon ,t_{ \alpha })= \int _{\Upsilon } \sum_{j=1}^{n} b_{j} \chi _{ \kappa _{j}}(\upsilon )\,d\mu ^{\blacktriangle }( \upsilon ,t_{\alpha }) = \ast _{j=1}^{n}\mu ^{\blacktriangle } \biggl(\kappa _{j}, \frac{t_{\alpha }}{b_{j}} \biggr). \end{aligned}$$

In [4] the authors showed:

(i) $\mu ^{\blacktriangle } (\kappa ,\frac{t_{\alpha }}{b} ) \ast \mu ^{\blacktriangle } (\kappa ,\frac{t_{\alpha }}{c} ) \leq \mu ^{\blacktriangle } (\kappa ,\frac{t_{\alpha }}{b+c} )$ for each $b,c\in \mathbb{R}^{+}$, $t_{\alpha } \in J^{o}$;
(ii) $\mu ^{\blacktriangle }: (\kappa ,\cdot ) :(0, +\infty ) \rightarrow I$ is continuous and increasing;
(iii) $\lim_{t_{\alpha }\longrightarrow 0}\mu ^{\blacktriangle }( \kappa ,t_{\alpha })=0$ and $\lim_{t_{\alpha }\longrightarrow +\infty } \mu ^{\blacktriangle }(\kappa ,t_{\alpha })=1$;
(iv) $\lim_{(t_{\alpha })_{n}\longrightarrow (t_{\alpha })_{0}}( \ast _{j=1}^{l}\mu ^{\blacktriangle }(\kappa _{j},(t_{\alpha })_{n}))= \ast _{j=1}^{l}\lim_{(t_{\alpha })_{n}\longrightarrow (t_{ \alpha })_{0}}\mu ^{\blacktriangle }(\kappa _{j},(t_{\alpha })_{n})$ for every $\kappa _{j}\cap \kappa _{i}=\emptyset $, $j\neq i$;
(v) $\lim_{(t_{\alpha })_{n}\longrightarrow (t_{\alpha })_{0}} \lim_{m\longrightarrow +\infty } (\mu ^{ \blacktriangle }(\kappa _{m},\frac{(t_{\alpha })_{n}}{a_{m}}))=\lim_{m\longrightarrow +\infty } \lim_{(t_{ \alpha })_{n}\longrightarrow (t_{\alpha })_{0}}(\mu ^{\blacktriangle } ( \kappa _{m},\frac{(t_{\alpha })_{n}}{a_{m}}))$.

In this article we let

(i)′: $\mu ^{\blacktriangle } (\kappa ,\frac{t_{\alpha }}{b+c} ) = \mu ^{\blacktriangle } (\kappa ,\frac{t_{\alpha }}{b} ) \ast \mu ^{\blacktriangle } (\kappa ,\frac{t_{\alpha }}{c} )$ for each $b,c,t_{\alpha }\in J^{o}$.

If $\kappa \in \mathcal{M}$, then $\psi \chi _{\kappa }$ is a simple function $(\psi \chi _{\kappa }(\upsilon )= \sum_{j=1}^{n} b_{j} \chi _{\kappa \cap \omega _{j}} (\upsilon ) )$, so

$$\begin{aligned} \int _{\kappa } \psi (\upsilon )\,d\mu ^{\blacktriangle }(\upsilon ,t_{ \alpha }) = \int \psi \chi _{\kappa }(\upsilon )\,d\mu ^{\blacktriangle }( \upsilon ,t_{\alpha }). \end{aligned}$$

### Theorem 4.2

([4])

*Let* $\psi ^{\prime }(\upsilon )=\sum_{j=1}^{n}b_{j}\chi _{\omega _{j}}( \upsilon )$ *and* $\psi ^{\shortparallel }(\upsilon )=\sum_{i=1}^{m}c_{k}\chi _{ \kappa _{i}}(\upsilon )$ *be simple functions in* $L_{+}$. *Then*,

(i) $\int _{\Upsilon }0\,d\mu ^{\blacktriangle }(\upsilon ,t_{\alpha })=1$;
(ii) *If* $\psi ^{\prime }$ *and* $\psi ^{\shortparallel }$ *are simple functions and* $\psi ^{\prime },\psi ^{\shortparallel }\in L_{+}$, *then*
  $$\begin{aligned} \int \bigl(\psi ^{\prime }+\psi ^{\shortparallel } \bigr) (\upsilon )\,d\mu ^{ \blacktriangle }(\upsilon ,t_{\alpha }) = \biggl( \int \psi ^{\prime }( \upsilon )\,d\mu ^{\blacktriangle }(\upsilon ,t_{\alpha }) \biggr) \ast \biggl( \int \psi ^{\shortparallel }(\upsilon )\,d\mu ^{\blacktriangle }( \upsilon ,t_{\alpha }) \biggr); \end{aligned}$$
(iii) $\int b\psi ^{\prime }(\upsilon )\,d\mu ^{\blacktriangle }(\upsilon ,t_{ \alpha }) = \int \psi ^{\prime }(\upsilon )\,d\mu ^{\blacktriangle }( \upsilon ,\frac{t_{\alpha }}{b})$, *for all* $b>0$;
(iv) *If* $\psi ^{\prime }\leq \psi ''$, *then* $\int _{\Upsilon }\psi ^{\prime }(\upsilon )\,d\mu ^{\blacktriangle }( \upsilon ,t_{\alpha }) \geq \int _{\Upsilon }\psi ''(\upsilon )\,d\mu ^{ \blacktriangle }(\upsilon ,t_{\alpha })$;
(v) *The map*
  $$\begin{aligned}& \xi : \mathcal{M}\longrightarrow [0,1]{,} \\& \kappa \longrightarrow \int _{\kappa }\psi ^{\prime }(\upsilon )\,d\mu ^{ \blacktriangle }(\upsilon ,t_{\alpha }) \end{aligned}$$
  *is a* ∗-*FM on* $\mathcal{M}$, *for every* $t_{\alpha }>0$.

Now, we study the concept of ∗-fuzzy integral in $L_{+}$.

### Definition 4.3

If $\xi :\Upsilon \longrightarrow \mathbb{R}^{+}$ is a measurable function in $L_{+}$, we set

$$\begin{aligned} \int _{\Upsilon }\xi (\upsilon )\,d\mu ^{\blacktriangle }(\upsilon ,t_{ \alpha })=\inf \biggl\lbrace \int _{\Upsilon }\psi ^{\prime }(\upsilon )\,d\mu ^{\blacktriangle }(\upsilon ,t_{\alpha })\Bigm| 0\leq \psi ^{ \prime } \leq \xi , \psi ^{\prime } \text{ is a simple function} \biggr\rbrace . \end{aligned}$$

It is straightforward to prove that

(i) If $\xi ,\zeta \in L_{+}$ and $\xi \leq \zeta $ then $\int \xi (\upsilon )\,d\mu ^{\blacktriangle }(\upsilon ,t_{\alpha }) \geq \int \zeta (\upsilon )\,d\mu ^{\blacktriangle }(\upsilon ,t_{\alpha })$;
(ii) $\int b\xi (\upsilon )\,d\mu ^{\blacktriangle }(\upsilon ,t_{\alpha })= \int \xi (\upsilon )\,d\mu ^{\blacktriangle }(\upsilon , \frac{t_{\alpha }}{b})$, for all $t_{\alpha },b\in J^{o}$;
(iii) $\int (\xi +\zeta )(\upsilon )\,d\mu ^{\blacktriangle }(\upsilon ,t_{ \alpha })=(\int \xi (\upsilon )\,d\mu ^{\blacktriangle }(\upsilon ,t_{ \alpha }))\ast (\int \zeta (\upsilon )\,d\mu ^{\blacktriangle }(\upsilon ,t_{ \alpha }))$.

### Definition 4.4

Let $\xi :\Upsilon \longrightarrow \mathbb{R}^{+}$ be a measurable function in $L_{+}$. If $\int \xi (\upsilon )\,d\mu ^{\blacktriangle }(\xi ,t_{\alpha })>0$ then we say that *ξ* is fuzzy integrable. Also, if $(\Upsilon ,\mathcal{M},\mu ^{\blacktriangle },\ast )$ is a ∗-FMS, we set

$$\begin{aligned} L^{+}= \biggl\lbrace \xi :\Upsilon \longrightarrow \mathbb{R}^{+} : \int _{\Upsilon } \xi (\upsilon )\,d\mu ^{\blacktriangle }(\upsilon ,t_{ \alpha })>0 \biggr\rbrace . \end{aligned}$$

### Theorem 4.5

([4] Monotone convergence theorem)

*Let* $\lbrace \xi _{n}\rbrace \subset L_{+}$ *be a sequence such that* $\xi _{n}\leq \xi _{n+1}$ *for every* $n\in \mathbb{N}$ *and* $\xi =\lim_{n\rightarrow +\infty } \xi _{n}= \sup_{n\in \mathbb{N}}\lbrace \xi _{n}\rbrace $, *then*

$$\begin{aligned} \int _{\Upsilon } \xi (\upsilon )\,d\mu ^{\blacktriangle }(\upsilon ,t_{ \alpha })=\lim_{n\rightarrow +\infty } \int _{ \Upsilon } \xi _{n}(\upsilon )\,d\mu ^{\blacktriangle }(\upsilon ,t_{ \alpha }). \end{aligned}$$

### Lemma 4.6

([4] Fatou’s lemma)

*If* $\lbrace \xi _{n} \rbrace \subset L_{+}$ *is a sequence*, *then*

$$\begin{aligned} \int _{\Upsilon }\liminf_{n\rightarrow +\infty } \xi _{n}(\upsilon )\,d\mu ^{\blacktriangle }( \upsilon ,t_{\alpha }) \geq \liminf_{n\rightarrow +\infty } \int _{\Upsilon }\xi _{n}(\upsilon )\,d\mu ^{ \blacktriangle }(\upsilon ,t_{\alpha }). \end{aligned}$$

### Theorem 4.7

([4] Fundamental convergence theorem)

*Let* $\lbrace \xi _{n} \rbrace \subset L^{+}$ *be a sequence and* $\xi _{n} \longrightarrow \xi $ *a*.*e*., *then* $\xi \in L^{+}$ *and* $\int _{\Upsilon } \xi (\upsilon )\,d\mu ^{\blacktriangle }(\upsilon ,t_{ \alpha }) = \lim_{n\rightarrow +\infty } \int _{\Upsilon } \xi _{n}(\upsilon )\,d\mu ^{\blacktriangle }(\upsilon ,t_{ \alpha })$.

In [4] the authors proved that, if $1\leq p <+\infty $, then $(L^{+})^{p}$ is a complete ∗-fNLS. Also, they proved the following inequality.

### Theorem 4.8

(Hölder’s inequality)

*If* *ξ* *and* *ζ* *are measurable functions on* ϒ *and* $1< p<+\infty $ *such that* $\frac{1}{p}+\frac{1}{q}=1$, *then*

$$\begin{aligned} N^{\blacktriangle }_{1}(\xi \zeta ,t_{\alpha })\geq N_{p}^{ \blacktriangle } \bigl(\xi ,(p)^{\frac{1}{p}} t_{\alpha } \bigr) \ast N_{q}^{ \blacktriangle } \bigl(\zeta ,(q)^{\frac{1}{q}}t_{\alpha } \bigr). \end{aligned}$$

## Lebesgue–Radon–Nikodym theorem

### Definition 5.1

Let $(\Upsilon ,\mathcal{M},\mu ^{\blacktriangle },\ast )$ and $(\Upsilon ,\mathcal{M},\nu ^{\blacktriangle },\ast )$ be two ∗-FMSs. If $\nu ^{\blacktriangle }(\omega ,t_{\alpha })=1$ for each $\omega \in \mathcal{M}$ for which $\mu ^{\blacktriangle }(\omega ,t_{\alpha })=1$ then we say that $\nu ^{\blacktriangle }$ is absolutely continuous with respect to $\mu ^{\blacktriangle }$ and write $\nu ^{\blacktriangle }\ll \mu ^{\blacktriangle }$.

### Definition 5.2

Let $(\Upsilon ,\mathcal{M},\mu ^{\blacktriangle },\ast )$ and $(\Upsilon ,\mathcal{M},\nu ^{\blacktriangle },\ast )$ be two ∗-FMSs. If there exist $\omega ,\kappa \in \mathcal{M}$ such that $\omega \cap \kappa =\emptyset $, $\omega \cup \kappa =\Upsilon $, $\mu ^{\blacktriangle }(\omega ,t_{\alpha })=1$ and $\nu ^{\blacktriangle }(\kappa ,t_{\alpha })=1$ then we say $\mu ^{\blacktriangle }$, $\nu ^{\blacktriangle }$ are mutually singular or that $\nu ^{\blacktriangle }$ is singular with respect to $\mu ^{\blacktriangle }$ ($\nu ^{\blacktriangle }\perp \mu ^{ \blacktriangle }$).

Absolute continuity is in a sense the antithesis of mutual singularity. More precisely, if $\nu ^{\blacktriangle }\perp \mu ^{\blacktriangle }$ and $\nu ^{\blacktriangle }\ll \mu ^{\blacktriangle }$, then $\nu ^{\blacktriangle }=1$, for if *ω* and *κ* are disjoint sets such that $\omega \cup \kappa =\Upsilon $ and $\mu ^{\blacktriangle }(\omega ,t_{\alpha })=\nu ^{\blacktriangle }( \kappa ,t_{\alpha })=1$, then $\nu ^{\blacktriangle }\ll \mu ^{\blacktriangle }$ implies that $\nu ^{\blacktriangle }(\omega ,t_{\alpha })=1$ and $\nu ^{\blacktriangle }(\Upsilon ,t_{\alpha })=\nu ^{\blacktriangle }( \omega \cup \kappa ,t_{\alpha })=\nu ^{\blacktriangle }(\omega ,t_{ \alpha })\ast \nu ^{\blacktriangle }(\kappa ,t_{\alpha })=1$, then $\nu ^{\blacktriangle }=1$.

### Theorem 5.3

*Let* $(\Upsilon ,\mathcal{M},\mu ^{\blacktriangle },\ast )$ *and* $(\Upsilon ,\mathcal{M},\nu ^{\blacktriangle },\ast )$ *be two* ∗-*FMSs*. *Let* $\nu ^{\blacktriangle }$ *be a bounded* ∗-*FM*. *Then* $\nu ^{\blacktriangle }\ll \mu ^{\blacktriangle }$ *if and only if*

(*ED*): *for every* $0<\varepsilon ^{\prime }<1$ *there exists* $0<\delta ^{\prime }<1$ *such that* $\nu ^{\blacktriangle }(\omega ,t_{\alpha })>1-\varepsilon ^{\prime }$ *whenever* $\mu ^{\blacktriangle }(\omega ,t_{\alpha })>1-\delta ^{\prime }$.

### Proof

From condition $(ED)$ we conclude that $\nu ^{\blacktriangle }\ll \mu ^{\blacktriangle }$. On the other hand, if the condition $(ED)$ does not hold, then there exists $\varepsilon _{0}>0$ such that for each $j\in \mathbb{N}$ we can find $\omega _{j}\in \mathcal{M}$ with $\mu ^{\blacktriangle }(\omega _{j},t_{\alpha })>1-\frac{1}{2^{j}}$ and $\nu ^{\blacktriangle }(\omega _{j},t_{\alpha })\leq 1-\varepsilon _{0}$. Let $\kappa _{i}=\bigcup_{j=i}^{+\infty }\omega _{j}$ and $\kappa =\bigcap_{i=1}^{+\infty }\kappa _{i}$. Then,

5.1 $$\begin{aligned} \mu ^{\blacktriangle }(\kappa _{i},t_{\alpha }) &=\mu ^{\blacktriangle } \Biggl(\bigcup_{j=i}^{ +\infty } \omega _{j},t_{\alpha } \Biggr) \\ &\geq \ast _{j=i}^{+\infty }\mu ^{\blacktriangle }( \omega _{j},t_{\alpha }) \\ &\geq \ast _{j=i}^{+\infty } \biggl(1-\frac{1}{2^{j}} \biggr). \end{aligned}$$

Thus according to Theorem 2.6(iv) and (5.1),

$$\begin{aligned} \mu ^{\blacktriangle }(\kappa ,t_{\alpha }) &=\mu ^{\blacktriangle } \Biggl( \bigcap_{i=1}^{+\infty }\kappa _{i},t_{\alpha } \Biggr) \\ &=\lim_{i\rightarrow +\infty }\mu ^{ \blacktriangle }(\kappa _{i},t_{\alpha }) \\ &\geq \lim_{i\rightarrow +\infty }\ast _{j=i}^{ +\infty } \biggl(1-\frac{1}{2^{j}} \biggr) \\ &=1. \end{aligned}$$

Thus

$$\begin{aligned} \mu ^{\blacktriangle }(\kappa ,t_{\alpha })=1. \end{aligned}$$

But

$$\begin{aligned} \nu ^{\blacktriangle }(\kappa _{i},t_{\alpha }) &=\nu ^{\blacktriangle } \Biggl(\bigcup_{j=i}^{+\infty } \omega _{j},t \Biggr) \\ &\leq \nu ^{\blacktriangle }(\omega _{i},t_{\alpha }) \\ &\leq 1-\varepsilon _{0}. \end{aligned}$$

Since $\nu ^{\blacktriangle }(\kappa _{i},t_{\alpha })>0$, according to Theorem 2.6(iv), we get

$$\begin{aligned} 0< \nu ^{\blacktriangle }(\kappa ,t_{\alpha }) &=\nu ^{\blacktriangle } \Biggl(\bigcap_{i=1}^{+\infty }\kappa _{i},t_{\alpha } \Biggr) \\ &=\lim_{i\rightarrow {+\infty }}\nu ^{ \blacktriangle }(\kappa _{i},t_{\alpha }) < 1-\varepsilon _{0}. \end{aligned}$$

Thus, it is false that $\nu ^{\blacktriangle }\ll \mu ^{\blacktriangle }$. □

### Lemma 5.4

*Let* $\mu ^{\blacktriangle }$ *be a* ∗-*FM and* $\xi \in L_{+}$. *Then the* ∗-*FM* $\nu ^{\blacktriangle }$ *defined by* $\nu ^{\blacktriangle }(\omega ,t_{\alpha })=\int _{\omega } \xi ( \upsilon )\,d\mu ^{\blacktriangle }(\upsilon ,t_{\alpha })$ *is absolutely continuous with respect to* $\mu ^{\blacktriangle }$. *Also*, $\nu ^{\blacktriangle }$ *is a bounded* ∗-*FM if and only if* $\xi \in L^{+}(\mu ^{\blacktriangle })$.

### Proof

Let $\mu ^{\blacktriangle }(\omega ,t_{\alpha })=1$, so

$$\begin{aligned} \nu ^{\blacktriangle }(\omega ,t_{\alpha }) &= \int _{\omega }\xi ( \upsilon )\,d\mu ^{\blacktriangle }(\upsilon ,t_{\alpha }) \\ &= \int \xi \chi _{\omega }(\upsilon )\,d\mu ^{\blacktriangle }(\upsilon ,t_{ \alpha }) \\ &=\lim_{n\rightarrow +\infty } \biggl\lbrace \int \psi _{n}\chi _{\omega }(\upsilon )\,d\mu ^{\blacktriangle }( \upsilon ,t_{\alpha }): 0< \psi _{n}\leq \xi \text{and} \psi _{n} \text{ is simple} \biggr\rbrace \\ &=\lim_{n\rightarrow +\infty } \biggl(\ast _{j(n)=1}^{i(n)} \mu ^{\blacktriangle } \biggl(E\cap \omega _{n,j(n)}, \frac{t_{\alpha }}{b_{j(n)}} \biggr) \biggr) \\ &\geq \lim_{n\rightarrow +\infty } \biggl( \ast _{j(n)=1}^{i(n)} \mu ^{\blacktriangle } \biggl(\omega , \frac{t_{\alpha }}{b_{j(n)}} \biggr) \biggr) \\ &=1. \end{aligned}$$

Thus $\nu ^{\blacktriangle }\ll \mu ^{\blacktriangle }$.Now, if $\nu ^{\blacktriangle }(\Upsilon ,t_{\alpha })>0$ then $\nu ^{\blacktriangle }(\Upsilon ,t_{\alpha })=\int \xi (\upsilon )\,d\mu ^{\blacktriangle }(\upsilon ,t_{\alpha })>0$ so $\xi \in L^{+}$. Conversely, if $\xi \in L^{+}$ then $\nu ^{\blacktriangle }(\Upsilon ,t_{\alpha })=\int \xi (\upsilon )\,d\mu ^{\blacktriangle }(\upsilon ,t_{\alpha })>0$. □

### Corollary 5.5

*If* $\xi \in L^{+}(\mu ^{\blacktriangle })$, *for every* $\varepsilon ^{\prime }>0$ *there exists* $\delta ^{\prime }>0$ *such that* $\int _{\omega }\xi (\upsilon )\,d\mu ^{\blacktriangle }(\upsilon ,t_{ \alpha })>1-\varepsilon ^{\prime }$ *whenever* $\mu ^{\blacktriangle }(\omega ,t_{\alpha })> 1-\delta ^{\prime }$.

### Proof

We set $\nu ^{\blacktriangle }(\omega )=\int _{\omega }\xi (\upsilon )\,d\mu ^{ \blacktriangle }(\upsilon ,t_{\alpha })$, thus $\nu ^{\blacktriangle }$ is a bounded ∗-FM. By Lemma 5.4, we get $\nu ^{\blacktriangle }\ll \mu ^{\blacktriangle }$. □

We are going to prove an important theorem in ∗-FMS.

### Theorem 5.6

(Lebesgue–Radon–Nikodym theorem)

*Let* $(\Upsilon ,\mathcal{M},\mu ^{\blacktriangle },\ast )$ *and* $(\Upsilon ,\mathcal{M},\nu ^{\blacktriangle },\ast )$ *be two* ∗-*FMSs*. *Let* $\nu ^{\blacktriangle }$, $\mu ^{\blacktriangle }$ *be* *σ*-*bounded* ∗-*FMs*, *then we can find a unique* *σ*-*bounded* ∗-*FM* $\rho ^{\blacktriangle }$ *such that* $\rho ^{\blacktriangle }\ll \mu ^{\blacktriangle }$. *Moreover*, *there is a* $\mu ^{\blacktriangle }$-*integrable function* $\xi :\Upsilon \longrightarrow \mathbb{R^{+}}$ *such that* $d\rho ^{\blacktriangle }=\xi \,d\mu ^{\blacktriangle }$.

### Proof

Case I: Suppose that $\nu ^{\blacktriangle }$ and $\mu ^{\blacktriangle }$ are bounded ∗-FM. Let $\kappa =\lbrace \xi :\Upsilon \longrightarrow [0, +\infty ): \int _{\omega } \xi (\upsilon )\,d\mu ^{ \blacktriangle }(\upsilon ,t_{\alpha }) \geq \nu ^{\blacktriangle }( \omega ,t_{\alpha })$ for all $\omega \in \mathcal{M}\rbrace $. Since $0\in \kappa $, *κ* is nonempty. Also, if $\xi ,\zeta \in \kappa $, thus $\sigma (\upsilon )=\max (\xi (\upsilon ),\zeta (\upsilon ))\in \kappa $ and if $A=\lbrace \upsilon :\xi (\upsilon )>\zeta (\upsilon )\rbrace $ for any $\omega \in \mathcal{M}$ we have

$$\begin{aligned} \int _{\omega } \sigma (\upsilon )\,d\mu ^{\blacktriangle }(\upsilon ,t_{ \alpha }) &= \int _{\omega \cap (A\cup A^{c})} \sigma (\upsilon )\,d\mu ^{ \blacktriangle }(\upsilon ,t_{\alpha }) \\ &= \int _{(\omega \cap A)\cup (\omega \cap A^{c})}\sigma (\upsilon )\,d\mu ^{\blacktriangle }(\upsilon ,t_{\alpha }) \\ &= \int (\chi _{(\omega \cap A)\cup (\omega \cap A^{c})} \cdot \sigma ) ( \upsilon )\,d\mu ^{\blacktriangle }(\upsilon ,t_{\alpha }) \\ &= \int (\chi _{(\omega \cap A)}\cdot \sigma +\chi _{(\omega \cap A^{c})} \cdot \sigma ) (\upsilon )\,d\mu ^{\blacktriangle }(\upsilon ,t_{\alpha }). \end{aligned}$$

By Theorem 4.2(ii), we get

$$\begin{aligned} \int _{\omega } \sigma (\upsilon )\,d\mu ^{\blacktriangle }(\upsilon ,t_{ \alpha })\geq \biggl( \int \chi _{\omega \cap A}\cdot \sigma (\upsilon )\,d\mu ^{\blacktriangle }(\upsilon ,t_{\alpha }) \biggr)\ast \biggl( \int \chi _{\omega \cap A^{c}}\cdot \sigma (\upsilon )\,d\mu ^{ \blacktriangle }(\upsilon ,t_{\alpha }) \biggr). \end{aligned}$$

Thus,

$$\begin{aligned} \int _{\omega }\sigma (\upsilon )\,d\mu ^{\blacktriangle }(\upsilon ,t_{ \alpha }) &\geq \biggl( \int _{\omega \cap A}\sigma (\upsilon )\,d\mu ^{ \blacktriangle }(\upsilon ,t_{\alpha }) \biggr)\ast \biggl( \int _{\omega \cap A^{c}}\sigma (\upsilon )\,d\mu ^{\blacktriangle }(\upsilon ,t_{ \alpha }) \biggr) \\ &= \biggl( \int _{\omega \cap A}\xi (\upsilon )\,d\mu ^{\blacktriangle }( \upsilon ,t_{\alpha }) \biggr)\ast \biggl( \int _{\omega \cap A^{c}}\zeta ( \upsilon )\,d\mu ^{\blacktriangle }(\upsilon ,t_{\alpha }) \biggr) \\ &\geq \nu ^{\blacktriangle }(\omega \cap A,t_{\alpha })\ast \nu ^{ \blacktriangle } \bigl(\omega \cap A^{c},t_{\alpha } \bigr) \\ &=\nu ^{\blacktriangle }(\omega ,t_{\alpha }). \end{aligned}$$

Let $b=\inf \lbrace \int \xi (\upsilon )\,d\mu ^{\blacktriangle }(\upsilon ,t_{ \alpha }) : \xi \in \kappa \rbrace $, $0<\nu ^{\blacktriangle }(\Upsilon )<1$ and choose a sequence $\lbrace \xi _{n}\rbrace \subseteq \kappa $ whenever $\int \xi _{n}(\upsilon )\,d\mu ^{\blacktriangle }(\upsilon ,t_{\alpha }) \longrightarrow b$. Let $\zeta _{n}=\max \lbrace \xi _{1},\dots ,\xi _{n}\rbrace $ and $\xi =\sup_{n\in \mathbb{N}} \xi _{n}$. Then $\zeta _{n}\in \kappa $, $\zeta _{n}$ increases pointwise to *ξ* and $\int \zeta _{n}(\upsilon )\,d\mu ^{\ast }(\upsilon ,t)\leq \int \xi _{n}( \upsilon )\,d\mu ^{\blacktriangle }(\upsilon ,t_{\alpha })$. It follows that $\lim_{ n\to +\infty }\int \zeta _{n}( \upsilon )\,d\mu ^{\blacktriangle }(\upsilon ,t_{\alpha })=b$ and hence, by monotone convergence theorem (Theorem 4.5), we get

$$\begin{aligned} \lim_{n\rightarrow +\infty } \int \zeta _{n}( \upsilon )\,d\mu ^{\blacktriangle }(\upsilon ,t_{\alpha })= \int \xi ( \upsilon )\,d\mu ^{\blacktriangle }(\upsilon ,t_{\alpha }) \leq \lim_{n\rightarrow +\infty } \int \xi _{n}( \upsilon )\,d\mu ^{\blacktriangle }(\upsilon ,t_{\alpha })=b, \end{aligned}$$

thus

5.2 $$\begin{aligned} \int \xi (\upsilon )\,d\mu ^{\blacktriangle }(\upsilon ,t_{\alpha }) \leq b. \end{aligned}$$

On the other hand, $\zeta _{n}\in \kappa $, and so

5.3 $$\begin{aligned} \lim_{{n\to +\infty }} \int \zeta _{n}( \upsilon )\,d\mu ^{\blacktriangle }(\upsilon ,t_{\alpha })= \int \xi ( \upsilon )\,d\mu ^{\blacktriangle }(\upsilon ,t_{\alpha }) \geq b. \end{aligned}$$

From (5.2) and (5.3), we have $\int \xi (\upsilon )\,d\mu ^{\blacktriangle }(\upsilon ,t_{\alpha })=b$ so $\xi \in \kappa $.

We set $\rho ^{\blacktriangle }(\omega ,t_{\alpha })=\int _{\omega }\xi ( \upsilon )\,d\mu ^{\blacktriangle }(\upsilon ,t_{\alpha })$ and get:

(i) $\rho ^{\blacktriangle }(\emptyset ,t_{\alpha })=\int _{\emptyset }\xi ( \upsilon )\,d\mu ^{\blacktriangle }(\upsilon ,t_{\alpha })=1$;
(ii) $$\begin{aligned} \rho ^{\blacktriangle } \Biggl(\bigcup_{j=1}^{+\infty } \omega _{j},t_{\alpha } \Biggr) &= \int _{\cup \omega _{j}}\xi (\upsilon )\,d\mu ^{\blacktriangle }(\upsilon ,t_{\alpha }) \\ &= \int (\xi \chi _{\cup \omega _{j}}) (\upsilon )\,d\mu ^{ \blacktriangle }( \upsilon ,t_{\alpha }) \\ &= \int \Biggl(\sum_{j=1}^{+\infty }\xi \chi _{\cup \omega _{j}} \Biggr) (\upsilon )\,d\mu ^{\blacktriangle }(\upsilon ,t_{ \alpha }) \\ &=\ast _{j=1}^{+\infty } \int (\xi \chi _{\omega _{j}}) ( \upsilon )\,d\mu ^{\blacktriangle }( \upsilon ,t_{\alpha }) \\ &=\ast _{j=1}^{+\infty } \int _{\omega _{j}}\xi ( \upsilon )\,d\mu ^{\blacktriangle }(\upsilon ,t_{\alpha }) \\ &=\ast _{j=1}^{+\infty }\rho ^{\blacktriangle }( \omega _{j},t_{\alpha }), \end{aligned}$$

whenever $\omega _{j}\cap \omega _{l}=\emptyset $ for $j\neq l$. Thus $\rho ^{\blacktriangle }$ is a ∗-FM. Also, $\rho ^{\blacktriangle }(\Upsilon ,t_{\alpha })=\int _{\Upsilon }\xi ( \upsilon )\,d\mu ^{\blacktriangle }(\upsilon ,t_{\alpha })=b$, thus $\rho ^{\blacktriangle }$ is a bounded ∗-FM.From $\rho ^{\blacktriangle }\ll \mu ^{\blacktriangle }$, $\int \xi (\upsilon )\,d\mu ^{\blacktriangle }(\upsilon ,t_{\alpha })=b>0$ and $\xi \in L^{+}$ and so, using Corollary 5.5 and Theorem 5.3, we get $\rho ^{\blacktriangle }\ll \mu ^{\blacktriangle }$.

Case II: Suppose that $\mu ^{\blacktriangle }$, $\nu ^{\blacktriangle }$ are *σ*-bounded ∗-FMs. Thus, ϒ is a countable disjoint union of $\mu ^{\blacktriangle }$-bounded sets and a countable disjoint union of $\nu ^{\blacktriangle }$-bounded sets. Taking intersection of these, we obtain a disjoint sequence $\lbrace A_{j}\rbrace \subseteq \mathcal{M}$ such that $\mu ^{\blacktriangle }(A_{j},t_{\alpha })$ and $\nu ^{\blacktriangle }(A_{j},t_{\alpha })$ are bounded for all *j* and $\Upsilon =\bigcup_{j=1}^{+\infty }A_{j}$. Define $\mu ^{\blacktriangle }_{j}(\omega )=\mu ^{\blacktriangle }(\omega \cap A_{j})$ and $\nu ^{\blacktriangle }_{j}(\omega )=\nu ^{\blacktriangle }(\omega \cap A_{j})$. We set $\xi =\sum_{j=1}^{+\infty }\xi _{j}$ and $\rho ^{\blacktriangle } =\ast _{j=1}^{+\infty }(\rho _{j})^{ \blacktriangle }$. We assert $\rho ^{\blacktriangle }\ll \mu ^{\blacktriangle }$ and $\rho ^{\blacktriangle }(\omega ,t_{\alpha })>1-\varepsilon ^{\prime }$ for every $0<\varepsilon ^{\prime }<1$, thus $\int _{\omega } \xi (\upsilon )\,d\mu ^{\blacktriangle }(\upsilon ,t_{ \alpha })>1-\varepsilon ^{\prime }$ and $\int _{\omega }\xi (\upsilon )\,d\mu ^{\blacktriangle }(\upsilon ,t_{ \alpha })=1$. On the other hand, we have

$$\begin{aligned} \int _{\omega }\xi (\upsilon )\,d\mu ^{\blacktriangle }(\upsilon ,t_{ \alpha }) ={}& \int _{\omega }\sum_{j=1}^{ +\infty } \xi _{j}(\upsilon )\,d\mu ^{\blacktriangle }( \upsilon ,t_{\alpha }) \\ ={}&1\quad {\text{if and only if}}\quad \sum_{j=1}^{ +\infty } \xi _{j}\chi _{\omega }=0\quad \text{a.e.}, \\ &{} {{\text{if and only if}}}\quad \xi _{j}\chi _{\omega }=0 \quad \text{a.e., for every } j\in \mathbb{N}, \\ & {}{{\text{if and only if}}}\quad \omega =\emptyset \quad \text{a.e.,} \end{aligned}$$

thus $\mu ^{\blacktriangle }(\omega ,t_{\alpha })=1$ and $\rho ^{\blacktriangle }\ll \mu ^{\blacktriangle }$. □

## The dual of $(L^{+})^{p}$

We remember that a linear operator $\xi :(\Upsilon ,N^{\blacktriangle },\ast )\longrightarrow (\Upsilon ,N^{ \blacktriangledown },\blacktriangledown )$ is said to be bounded if there exists a constant $a \in \mathbb{R}\setminus \lbrace 0 \rbrace $ such that, for all $\upsilon \in \Upsilon $ and $t_{\alpha }>0$,

$$\begin{aligned} N^{\blacktriangledown } \bigl(\xi (\upsilon ),t_{\alpha } \bigr) \geq N^{ \blacktriangle }(a \upsilon ,t_{\alpha }). \end{aligned}$$

### Theorem 6.1

*If* $N^{\blacktriangle }_{p}: ((L^{+})^{p})^{\ast } \times J^{o} \longrightarrow (0,1]$ *is a fuzzy set such that* $N_{p}^{\blacktriangle }(\phi ,t_{\alpha }) = \inf \lbrace N_{ \mathbb{R}}^{\blacktriangle }(\phi (\xi ),t_{\alpha })$: *for all* $\xi \in (L^{+})^{p}\rbrace $, *then* $((L^{+})^{p})^{\ast }$ *is a complete* ∗-*fNLS*.

### Proof

1. $N_{ p}^{\blacktriangle }(\phi ,t_{\alpha })\geq 0$ is obvious.
2. $\phi \neq0$ if and only if there exist $\xi \in (L^{+})^{p}$ such that $\phi (\xi )>0$, if and only if $N^{\blacktriangle }_{\mathbb{R}}(\phi (\xi ),t_{\alpha })\neq1$, if and only if $N^{\blacktriangle }_{P}(\phi ,t_{\alpha })\neq1$.
3. $$\begin{aligned} N_{ p}^{\blacktriangle }(b\phi ,t_{\alpha }) &=\inf \bigl\lbrace N_{ \mathbb{R}}^{\blacktriangle } \bigl((b\phi ) (\xi ),t_{\alpha } \bigr):{ \text{for all }} \xi \in \bigl(L^{+} \bigr)^{p} \bigr\rbrace \\ &=\inf \biggl\lbrace N_{\mathbb{R}}^{\blacktriangle } \biggl(\phi (\xi ), \frac{t_{\alpha }}{b} \biggr):{\text{for all }} \xi \in \bigl(L^{+} \bigr)^{p} \biggr\rbrace \\ &=N^{\blacktriangle }_{p} \biggl(\phi ,\frac{t_{\alpha }}{b} \biggr), \end{aligned}$$
  for some $b>0$.
4. $$\begin{aligned} N_{p}^{\blacktriangle } \bigl(\phi ^{\prime }+\phi '',t_{\alpha }+t_{\beta } \bigr) &= \inf \bigl\lbrace N_{\mathbb{R}}^{\blacktriangle } \bigl( \bigl(\phi ^{\prime }+\phi '' \bigr) ( \xi ),t_{\alpha }+t_{\beta } \bigr) \bigr\rbrace \\ &=\inf \bigl\lbrace N_{\mathbb{R}}^{\blacktriangle } \bigl(\phi ^{\prime }(\xi )+ \phi ''(\xi ) \bigr),t_{\alpha }+t_{\beta }) \bigr\rbrace \\ &\geq \inf \bigl\lbrace N_{\mathbb{R}}^{\blacktriangle } \bigl(\phi ^{\prime }( \xi ),t_{\alpha } \bigr)\ast N_{\mathbb{R}}^{\blacktriangle } \bigl(\phi ''(\xi ),t_{ \beta } \bigr) \bigr\rbrace \\ &\geq \inf \bigl\lbrace N_{\mathbb{R}}^{\blacktriangle } \bigl(\phi ^{\prime }( \xi ),t_{\alpha } \bigr) \bigr\rbrace \ast \inf \bigl\lbrace N_{\mathbb{R}}^{ \blacktriangle } \bigl(\phi ''( \xi ),t_{\beta } \bigr) \bigr\rbrace \\ &=N_{p}^{\blacktriangle } \bigl(\phi ^{\prime },t_{\alpha } \bigr)\ast N_{p}^{ \blacktriangle } \bigl(\phi '',t_{\beta } \bigr). \end{aligned}$$
5. $$\begin{aligned} N_{p}^{\blacktriangle }(\phi ,\cdot )=\inf \bigl\lbrace N_{\mathbb{R}}^{ \blacktriangle } \bigl(\phi (\xi ),\cdot \bigr):\xi \in \bigl(L^{+} \bigr)^{p} \bigr\rbrace , \end{aligned}$$
  $N_{\mathbb{R}}^{\blacktriangle }(\phi (\xi ),\cdot )$ is increasing and continuous in the second component, so $N_{p}^{\blacktriangle }(\phi ,\cdot )$.
6. $$\begin{aligned} \lim_{t_{\alpha }\rightarrow 0}N_{p}^{\blacktriangle }(\phi ,t_{ \alpha }) &=\lim_{t_{\alpha }\rightarrow 0} \bigl(\inf \bigl\lbrace N_{ \mathbb{R}}^{\blacktriangle } \bigl(\phi (\xi ),t_{\alpha } \bigr):{\text{for all }} \xi \in \bigl(L^{+} \bigr)^{p} \bigr\rbrace \bigr) \\ &=\inf \lim_{t_{\alpha }\rightarrow 0} \bigl\lbrace N_{\mathbb{R}}^{ \blacktriangle } \bigl(\phi (\xi ),t_{\alpha } \bigr):{\text{for all }} \xi \in \bigl(L^{+} \bigr)^{p} \bigr\rbrace \\ &=\inf \lbrace 0\rbrace \\ &=0 \end{aligned}$$
  and
  $$\begin{aligned} \lim_{t_{\alpha }\rightarrow +\infty }N_{p}^{ \blacktriangle }(\phi ,t_{\alpha })=1. \end{aligned}$$

Let $\lbrace \phi _{n}\rbrace \subseteq ((L^{+})^{p})^{\blacktriangle }$ be a Cauchy sequence. Then for every $\xi \in (L^{+})^{p}$, $\lbrace \phi _{n}(\xi )\rbrace \subseteq \mathbb{R}^{+}$ is a Cauchy sequence in $\mathbb{R}$ and, since $\mathbb{R}$ is complete, we can find $\upsilon _{\xi }\in \mathbb{R}^{+}$ such that $\phi _{n}(\xi )\longrightarrow \upsilon _{\xi }$. Now, we define $\phi :(L^{+})^{p}\longrightarrow \mathbb{R}^{+}$ by $\phi (\xi )=\upsilon _{\xi }$. Hence $\phi _{n}\longrightarrow \phi $ almost everywhere, also $\phi _{n}$ is linear, i.e., $\phi _{n}(b \xi +\zeta )=b\phi _{n}(\xi )+\phi _{n}(\zeta )$, and so

$$\begin{aligned} \phi (b \xi +\zeta ) &=\lim_{n\rightarrow +\infty }\phi _{n}(b \xi +\zeta ) \\ &=\lim_{n\rightarrow +\infty } \bigl(b\phi _{n}( \xi )+\phi _{n}(\zeta ) \bigr) \\ &=b\lim_{n\rightarrow +\infty }\phi _{n}( \xi )+\lim _{n\rightarrow +\infty }\phi _{n}( \zeta ) \\ &=b\phi (\xi )+\phi (\zeta ). \end{aligned}$$

From Lemma 3.2, we conclude that *ϕ* is fuzzy bounded and so $\phi \in ((L^{+})^{p})^{\ast }$. □

### Theorem 6.2

*Suppose that* *p* *and* *q* *are conjugate exponents and* $1\leq q<+\infty $. *If* $\zeta \in (L^{+})^{q}$ *then*

$$\begin{aligned} N_{q}^{\blacktriangle }(\zeta ,t_{\alpha })=N^{\blacktriangle }( \phi _{ \zeta },t_{\alpha })=\inf \biggl\lbrace \int \xi \zeta \,d\mu ^{ \blacktriangle }(\upsilon ,t_{\alpha }): N_{p}^{\blacktriangle }(\xi ,t_{ \alpha })\geq \frac{1}{b} \biggr\rbrace , \end{aligned}$$

*for some constant* $b\in (1,+\infty )$.

### Proof

We have $N_{p}^{\blacktriangle }(\xi ,t_{\alpha })\geq \frac{1}{b}$ so $\int (\xi (\upsilon ))^{p}\,d\mu ^{\blacktriangle }(\upsilon ,t_{\alpha }) \geq \frac{1}{b}>0$. It means $\xi \in (L^{+})^{p}$. By Hölder’s inequality, we get

$$\begin{aligned} N_{1}^{\blacktriangle }(\xi \zeta ,t_{\alpha })\geq \bigl(N_{p}^{ \blacktriangle } \bigl(\xi ,(p)^{\frac{1}{p}}\cdot t_{\alpha } \bigr) \bigr) \ast \bigl(N_{q}^{\blacktriangle } \bigl(\zeta ,(q)^{\frac{1}{q}}\cdot t_{\alpha } \bigr) \bigr). \end{aligned}$$

We have $p^{\frac{1}{p}}>1$ and $q^{\frac{1}{q}}>1$, thus

$$\begin{aligned} N_{1}^{\blacktriangle }(\xi \zeta ,t_{\alpha }) &\geq \bigl(N_{p}^{ \blacktriangle } \bigl(\xi ,(p)^{\frac{1}{p}}\cdot t_{\alpha } \bigr) \bigr) \ast \bigl(N_{q}^{\blacktriangle } \bigl(\zeta ,(q)^{\frac{1}{q}}\cdot t_{\alpha } \bigr) \bigr) \\ &\geq \bigl(N_{p}^{\blacktriangle }(\xi ,t_{\alpha }) \bigr) \ast \bigl(N_{q}^{ \blacktriangle }(\zeta ,t_{\alpha }) \bigr) \\ &\geq \frac{1}{b}\ast N_{q}^{\blacktriangle }(\zeta ,t_{\alpha }) \end{aligned}$$

and

6.1 $$\begin{aligned} N^{\blacktriangle }(\phi _{\xi },t_{\alpha }) &= \inf \biggl\lbrace N_{1}^{\blacktriangle }(\xi \zeta ,t_{\alpha }): N_{p}^{ \blacktriangle }(\xi ,t_{\alpha }) \geq \frac{1}{b} \biggr\rbrace \\ &\geq \frac{1}{b}\ast N_{q}^{\blacktriangle }(\zeta ,t_{\alpha }). \end{aligned}$$

When $b\longrightarrow 1$, we have

$$\begin{aligned} N^{\blacktriangle }(\phi _{\zeta },t_{\alpha })\geq \lim _{b \rightarrow 1}\frac{1}{b}\ast \lim_{b\rightarrow 1}N_{q}^{ \blacktriangle }( \zeta ,t_{\alpha }), \end{aligned}$$

and then

6.2 $$\begin{aligned} N^{\blacktriangle }(\phi _{\zeta },t_{\alpha }) \geq N_{q}^{ \blacktriangle }(\zeta ,t_{\alpha }). \end{aligned}$$

Let $\xi =\zeta ^{(q-1)}$. Then

$$\begin{aligned} \int \bigl(\xi (\upsilon ) \bigr)^{p}\,d\mu ^{\blacktriangle }( \upsilon ,t_{\alpha }) &= \int \bigl(\zeta (\upsilon ) \bigr)^{(pq-p)}\,d\mu ^{\blacktriangle }( \upsilon ,t_{ \alpha }) \\ &= \int \bigl(\zeta (\upsilon ) \bigr)^{q}\,d\mu ^{\blacktriangle }( \upsilon ,t_{ \alpha })>0 \end{aligned}$$

and $\xi \in (L^{+})^{p}$. Since

$$\begin{aligned} N^{\blacktriangle }(\phi _{\zeta },t_{\alpha }) &\leq \int \xi \zeta ( \upsilon )\,d\mu ^{\blacktriangle }(\upsilon ,t_{\alpha }) \\ &= \int \bigl(\zeta (\upsilon ) \bigr)^{q}\,d\mu ^{\blacktriangle }( \upsilon ,t_{ \alpha }) \\ &=N_{q}^{\blacktriangle }(\zeta ,t_{\alpha }), \end{aligned}$$

we get

6.3 $$\begin{aligned} N^{\blacktriangle }(\phi _{\zeta },t_{\alpha }) \leq N_{q}^{ \blacktriangle }(\zeta ,t_{\alpha }). \end{aligned}$$

From (6.2) and (6.3), we conclude that

$$\begin{aligned} N^{\blacktriangle }(\phi _{\zeta },t_{\alpha })=N_{q}^{\blacktriangle }( \zeta ,t_{\alpha }). \end{aligned}$$

 □

Conversely, if $\xi \longrightarrow \int \xi \zeta (\upsilon )\,d\mu ^{\blacktriangle }( \upsilon ,t_{\alpha })$ is a bounded linear functional on $(L^{+})^{p}$, then $\zeta \in (L^{+})^{q}$.

### Theorem 6.3

*Consider the conjugate exponents* *p* *and* *q* *in which* $q<+\infty $. *Let* *ζ* *be a measurable function on* ϒ *such that* $\xi \zeta \in (L^{+})^{1}$ *for all* *ξ* *in* Σ *of simple functions that vanish outside a set of bounded* ∗-*FM*. *For some constant* $b>1$, *define*

$$\begin{aligned} M_{q}(\zeta ,t_{\alpha })=\inf \biggl\lbrace \int (\xi \zeta ) ( \upsilon )\,d\mu ^{\blacktriangle }(\upsilon ,t_{\alpha }): \xi \in \Sigma , N_{p}^{\blacktriangle }(\xi ,t_{\alpha })\geq \frac{1}{b} \biggr\rbrace >0 \end{aligned}$$

*and assume that either* $S_{\zeta }=\lbrace \upsilon : \zeta (\upsilon )\neq0\rbrace $ *is* *σ*-*bounded or* $\mu ^{\blacktriangle }$ *is* ∗-*fuzzy bounded*. *Then* $\zeta \in (L^{+})^{q}$ *and* $M_{q}(\zeta ,t_{\alpha })=N_{q}^{\blacktriangle }(\zeta ,t_{\alpha })$.

### Proof

If *ξ* is a bounded measurable function that vanishes outside a set *ω* of bounded ∗-FM and $N_{p}^{\blacktriangle }(\xi ,t_{\alpha })\geq \frac{1}{b}$ for some ${b\in (1,+\infty )}$, then $\int (\xi \zeta )(\upsilon )\,d\mu ^{\blacktriangle }(\upsilon ,t_{ \alpha })\leq M_{q}(\zeta ,t_{\alpha })$. Indeed, by Theorem 3.4, we can find a sequence $\lbrace \xi _{n}\rbrace $ of simple functions such that $\xi _{n}\uparrow \xi $ a.e. Thus $\xi _{n}\zeta \uparrow \xi \zeta $. By the fundamental theorem (Theorem 4.7), we get

$$\begin{aligned} \int (\xi \zeta ) (\upsilon )\,d\mu ^{\blacktriangle }(\upsilon ,t_{ \alpha }) &=\lim_{n\rightarrow +\infty } \int (\xi _{n} \zeta ) (\upsilon )\,d\mu ^{\blacktriangle }( \upsilon ,t_{\alpha }) \\ &\geq \inf \biggl\lbrace \int (\xi \zeta ) (\upsilon )\,d\mu ^{ \blacktriangle }(\upsilon ,t_{\alpha }): \xi \in \Sigma , N_{p}^{ \blacktriangle }(\xi ,t_{\alpha })\geq \frac{1}{b} \biggr\rbrace \\ &=M_{q}(\zeta ,t_{\alpha }) \end{aligned}$$

and

6.4 $$\begin{aligned} \int (\xi \zeta ) (\upsilon )\,d\mu ^{\blacktriangle }(\upsilon ,t_{ \alpha })\geq M_{q}(\zeta ,t_{\alpha }). \end{aligned}$$

Now, we suppose that ${q<+\infty }$ and $S_{\zeta }$ is *σ*-bounded. Let $\lbrace \omega _{n}\rbrace $ be an increasing sequence of sets such that $\mu ^{\blacktriangle }(\omega _{n},t_{\alpha })>0$ for every $n\in \mathbb{N}$, thus $S_{\zeta }=\bigcup_{n=1}^{+\infty }\omega _{n}$. Let $\lbrace \phi _{n}\rbrace $ be a sequence of simple functions such that $\phi _{n}\uparrow \zeta $ pointwise and let $\zeta _{n}=\phi _{n}\chi _{\omega _{n}}$. Then $\zeta _{n}\uparrow \zeta $ pointwise and $\zeta _{n}$ vanishes outside $\omega _{n}$.

Let $\xi _{n}=\zeta _{n}^{q-1}$, then by Fatou’s lemma, we get

6.5 $$\begin{aligned} \int \lim_{n\rightarrow +\infty } \inf (\xi _{n} \zeta _{n}) (\upsilon )\,d\mu ^{\blacktriangle }(\upsilon ,t_{\alpha }) &\geq \lim_{n\rightarrow +\infty }\inf \int ( \xi _{n}\zeta _{n}) (\upsilon )\,d\mu ^{\blacktriangle }(\upsilon ,t_{ \alpha }) \\ &\geq \lim_{n\rightarrow +\infty }\inf \int ( \xi _{n}\zeta ) (\upsilon )\,d\mu ^{\blacktriangle }( \upsilon ,t_{\alpha }) \end{aligned}$$

and

$$\begin{aligned} \int \lim_{n\rightarrow +\infty }\inf (\xi _{n} \zeta _{n}) (\upsilon )\,d\mu ^{\blacktriangle }(\upsilon ,t_{\alpha }) &= \int \lim_{n\rightarrow +\infty }\inf \bigl( \zeta _{n}( \upsilon ) \bigr)^{q}\,d\mu ^{\blacktriangle }(\upsilon ,t_{\alpha }) \\ &= \int \bigl(\zeta (\upsilon ) \bigr)^{q}\,d\mu ^{\blacktriangle }( \upsilon ,t_{ \alpha }) \\ &=N_{q}^{\blacktriangle }(\zeta ,t_{\alpha }). \end{aligned}$$

From (6.5), we get

$$\begin{aligned} N_{q}^{\blacktriangle } (\zeta ,t_{\alpha }) > \lim _{n \rightarrow +\infty } \inf \int (\xi _{n}\zeta ) ( \upsilon )\,d\mu ^{\blacktriangle }( \upsilon ,t_{\alpha }) \end{aligned}$$

and, by (6.4), we get

6.6 $$\begin{aligned} N_{q}^{\blacktriangle }(\zeta ,t_{\alpha })\geq M_{q}(\zeta ,t_{\alpha }). \end{aligned}$$

On the other hand, using Hölder’s inequality, we get

$$\begin{aligned} \int (\xi \zeta ) (\upsilon )\,d\mu ^{\blacktriangle }(\upsilon ,t_{ \alpha }) &\geq \bigl(N_{p}^{\blacktriangle } \bigl(\xi ,(p)^{\frac{1}{p}}\cdot t_{ \alpha } \bigr) \bigr)\ast \bigl(N_{q}^{\blacktriangle } \bigl(\zeta ,(q)^{\frac{1}{q}}\cdot t_{ \alpha } \bigr) \bigr) \\ &\geq \bigl(N_{p}^{\blacktriangle }(\xi ,t_{\alpha }) \bigr) \ast \bigl(N_{q}^{ \blacktriangle }(\zeta ,t_{\alpha }) \bigr) \\ &\geq \frac{1}{b} \ast \bigl(N_{q}^{\blacktriangle }(\zeta ,t_{\alpha }) \bigr). \end{aligned}$$

Letting *b* to 1, we get

$$\begin{aligned} \int (\xi \zeta ) (\upsilon )\,d\mu ^{\blacktriangle }(\upsilon ,t_{ \alpha }) &\geq \lim_{b\rightarrow 1} \biggl( \frac{1}{b} \ast \bigl(N_{q}^{ \blacktriangle }(\zeta ,t_{\alpha }) \bigr) \biggr) \\ &\geq \lim_{b\rightarrow 1}\frac{1}{b}\ast \lim _{b \rightarrow 1}N_{q}^{\blacktriangle }(\zeta ,t_{\alpha }). \end{aligned}$$

Thus,

$$\begin{aligned} \int (\xi \zeta ) (\upsilon )\,d\mu ^{\blacktriangle }(\upsilon ,t_{ \alpha })\geq N_{q}^{\blacktriangle }(\zeta ,t_{\alpha }) \end{aligned}$$

and

$$\begin{aligned} \inf \int (\xi \zeta ) (\upsilon )\,d\mu ^{\blacktriangle }(\upsilon ,t_{ \alpha })\geq N_{q}^{\blacktriangle }(\zeta ,t_{\alpha }), \end{aligned}$$

and then

6.7 $$\begin{aligned} M_{q}(\zeta ,t_{\alpha })\geq N_{q}^{\blacktriangle }(\zeta ,t_{\alpha }). \end{aligned}$$

Now, (6.6), (6.7), and $M_{q}(\zeta ,t_{\alpha })=N_{q}^{\blacktriangle }(\zeta ,t_{\alpha })>0$, imply that $\zeta \in (L^{+})^{q}$ when $q<+\infty $. □

Now, we study the surjectivity of the map $\phi \longrightarrow \phi _{\zeta }$ in $((L^{+})^{p})^{\ast }$.

### Theorem 6.4

*Consider the conjugate exponents* *p* *and* *q* *and let* ${1< p<+\infty }$. *For each* $\phi ^{\prime }\in ((L^{+})^{p})^{\ast }$, *we can find* $\zeta \in (L^{+})^{q}$ *such that* $\int \phi ^{\prime }(\xi )\,d\mu ^{\blacktriangle }(\upsilon ,t_{\alpha })= \int (\xi \zeta )(\upsilon )\,d\mu ^{\blacktriangle }(\upsilon ,t_{ \alpha })$ *for all* $\xi \in (L^{+})^{p}$ *and hence* $(L^{+})^{q}$ *is isometrically isomorphic to* $((L^{+})^{p})^{\ast }$.

### Proof

First, let us suppose that $\mu ^{\blacktriangle }$ is a bounded ∗-FM $(\mu ^{\blacktriangle }(\Upsilon ,t_{\alpha })>0)$. Suppose that $\phi ^{\prime }\in ((L^{+})^{p})^{\ast }$ and *ω* is a ∗-FM set. Also let $\nu ^{\blacktriangle }(\omega ,t_{\alpha })=\int \phi ^{\prime }(\chi _{ \omega })(\upsilon )\,d\mu ^{\blacktriangle }(\upsilon ,t_{\alpha })$. Then, we have

(i) $$\begin{aligned} \nu ^{\blacktriangle }(\emptyset ,t) &= \int \phi ^{\prime } \bigl(\chi _{\phi }( \upsilon ) \bigr)\,d\mu ^{\blacktriangle }(\upsilon ,t_{\alpha }) \\ &= \int \phi ^{\prime }(0)\,d\mu ^{\blacktriangle }(\upsilon ,t_{\alpha }) \\ &= \int 0\,d\mu ^{\blacktriangle }(\upsilon ,t_{\alpha }) \\ &=1. \end{aligned}$$
(ii) $$\begin{aligned} \nu ^{\blacktriangle } \Biggl(\bigcup_{j=1}^{ +\infty } \omega _{j} \Biggr) &= \int \phi ^{\prime } (\chi _{\bigcup _{j=1}^{+\infty } \omega _{j}} )\,d\mu ^{\blacktriangle }(\upsilon ,t_{\alpha }) \\ &= \int \phi ^{\prime } \Biggl(\sum_{j=1}^{+\infty } \chi _{\omega _{j}} \Biggr)\,d\mu ^{\blacktriangle }(\upsilon ,t_{\alpha }) \\ &= \int \sum_{j=1}^{+\infty }\phi ^{\prime }(\chi _{ \omega _{j}})\,d\mu ^{\blacktriangle }(\upsilon ,t_{\alpha }) \\ &=\ast _{j=1}^{+\infty }\nu ^{\blacktriangle }(\omega _{j},t_{ \alpha }), \end{aligned}$$

whenever $\omega _{j}\cap \omega _{l}=\emptyset $, $j\neq l$. Hence, $\nu ^{\blacktriangle }$ is a ∗-FM. Also, if $\mu ^{\blacktriangle }(\omega ,t_{\alpha })=1$, then $\mu ^{\blacktriangle } (\omega ,t_{\alpha })=\int \chi _{\omega }\,d\mu ^{ \blacktriangle }(\upsilon ,t_{\alpha })=1$ hence $\chi _{\omega }=0$ a.e. as an element of $(L^{+})^{p}$. Thus

$$\begin{aligned} \nu ^{\blacktriangle }(\omega ,t_{\alpha }) &= \int \phi ^{\prime }(\chi _{ \omega })\,d\mu ^{\blacktriangle }( \upsilon ,t_{\alpha }) \\ &= \int 0 \,d\mu ^{\blacktriangle }(\upsilon ,t_{\alpha }) \\ &=1, \end{aligned}$$

which implies that $\nu ^{\blacktriangle }\ll \mu ^{\blacktriangle }$. Using the Lebesque–Radon–Nikodym theorem (Theorem 5.6), we can find $\zeta \in L^{+}$ such that

6.8 $$\begin{aligned} \int \phi ^{\prime }(\chi _{\omega })\,d\mu ^{\blacktriangle }( \upsilon ,t_{ \alpha })=\nu ^{\blacktriangle }(\omega ,t_{\alpha })= \int _{\omega } \zeta (\upsilon )\,d\mu ^{\blacktriangle }(\upsilon ,t_{\alpha }), \end{aligned}$$

for all *ω*. Now, if $\xi =\sum_{j=1}^{n}b_{j}\chi _{\omega _{j}}$ is a simple function, we have

$$\begin{aligned} \int \phi ^{\prime }(\xi )\,d\mu ^{\blacktriangle }(\upsilon ,t_{\alpha }) &= \int \phi ^{\prime } \Biggl(\sum_{j=1}^{n}b_{j} \chi _{\omega _{j}} \Biggr)\,d\mu ^{\blacktriangle }(\upsilon ,t_{\alpha }) \\ &= \int \sum_{j=1}^{n} \bigl(b_{j}\phi ^{\prime }(\chi _{\omega _{j}}) \bigr)\,d\mu ^{\blacktriangle }(\upsilon ,t_{\alpha }) \\ &=\ast _{j=1}^{n} \int b_{j}\phi ^{\prime }(\chi _{\omega _{j}})\,d\mu ^{ \blacktriangle }(\upsilon ,t_{\alpha }) \\ &=\ast _{j=1}^{n} \int \phi ^{\prime }(\chi _{\omega _{j}})\,d\mu ^{ \blacktriangle } \biggl(\upsilon ,\frac{t_{\alpha }}{ b_{j}} \biggr). \end{aligned}$$

From (6.8), we get

6.9 $$\begin{aligned} \int \phi ^{\prime }(\xi )\,d\mu ^{\blacktriangle }(\upsilon ,t_{\alpha })= \ast _{j=1}^{n} \int _{\omega _{j}}\zeta (\upsilon )\,d\mu ^{ \blacktriangle } \biggl( \upsilon ,\frac{t_{\alpha }}{b_{j}} \biggr). \end{aligned}$$

On the other hand,

6.10 $$\begin{aligned} \int (\xi \zeta ) (\upsilon )\,d\mu ^{\blacktriangle }(\upsilon ,t_{ \alpha }) &= \int \Biggl(\sum_{j=1}^{n}b_{j} \chi _{\omega _{j}} \Biggr) \cdot \zeta \,d\mu ^{\blacktriangle }(\upsilon ,t_{\alpha }) \\ &=\ast _{j=1}^{n} \int b_{j}(\chi _{\omega _{j}}\cdot \zeta ) ( \upsilon )\,d\mu ^{\blacktriangle }(\upsilon ,t_{\alpha }) \\ &=\ast _{j=1}^{n} \int (\chi _{\omega _{j}}\cdot \zeta ) (\upsilon )\,d\mu ^{\blacktriangle } \biggl(\upsilon ,\frac{t_{\alpha }}{b_{j}} \biggr) \\ &=\ast _{j=1}^{n} \int _{\omega _{j}}\zeta (\upsilon )\,d\mu ^{ \blacktriangle } \biggl( \upsilon ,\frac{t_{\alpha }}{ b_{j}} \biggr). \end{aligned}$$

Using (6.9) and (6.10), we get $\int \phi ^{\prime }(\xi )\,d\mu ^{\blacktriangle }(\upsilon ,t_{\alpha })= \int (\xi \zeta )(\upsilon )\,d\mu ^{\blacktriangle }(\upsilon ,t_{ \alpha })$ for all simple functions *ξ*. Moreover, $\phi ^{\prime }(\xi )\leq \Vert \phi ^{\prime }\Vert \cdot \Vert \xi \Vert $ thus,

$$\begin{aligned} \int \phi ^{\prime }(\xi )\,d\mu ^{\blacktriangle }(\upsilon ,t_{\alpha }) &= \int (\xi \zeta ) (\upsilon )\,d\mu ^{\blacktriangle }(\upsilon ,t_{ \alpha }) \\ &> \int _{\Upsilon } \bigl\Vert \phi ^{\prime } \bigr\Vert \cdot \Vert \xi \Vert d \mu ^{\blacktriangle }(\upsilon ,t_{\alpha }) \\ &=\mu ^{\blacktriangle } \biggl(\Upsilon , \frac{t_{\alpha }}{ \Vert \phi ^{\prime } \Vert \cdot \Vert \xi \Vert } \biggr)>0. \end{aligned}$$

By Theorem 6.3, we have $\zeta \in (L^{+})^{q}$.

Now, suppose that $\mu ^{\blacktriangle }$ is a *σ*-bounded ∗-FM. Let $\lbrace \omega _{n}\rbrace $ be an increasing sequence of sets such that $\mu ^{\blacktriangle }(\omega _{n},t_{\alpha })>0$ and $\Upsilon =\bigcup_{n=1}^{+\infty }\omega _{n}$, and let us agree to identify $(L^{+})^{p}(\omega _{n})$ and $(L^{+})^{q}(\omega _{n})$ with the subspaces of $(L^{+})^{p}(\Upsilon )$ and $(L^{+})^{q}(\Upsilon )$ consisting of functions that vanish outside $\omega _{n}$. The preceding argument shows that for each $n\in \mathbb{N}$ there exists $\zeta _{n}\in (L^{+})^{q}(\omega _{n})$ such that $\int \phi ^{\prime }(\xi )\,d\mu ^{\blacktriangle }(\upsilon ,t_{\alpha })= \int (\xi \zeta _{n})(\upsilon )\,d\mu ^{\blacktriangle }(\upsilon ,t_{ \alpha })$ for all $\xi \in (L^{+})^{p}(\omega _{n})$ and $N_{q}^{\blacktriangle }(\zeta _{n},t_{\alpha })=N^{\blacktriangle }( \phi ^{\prime }_{ \vert (L^{+})^{p} (\omega _{n})},t_{\alpha })> N^{ \blacktriangle }(\phi ^{\prime },t_{\alpha })$, $\phi ^{\prime }(\xi )=\int (\xi \zeta _{n})(\upsilon )\,d\mu ^{ \blacktriangle }(\upsilon ,t_{\alpha })$.The function $\zeta _{n}$ is unique on null sets. Thus $\zeta _{n}=\zeta _{m}$ a.e. On $\omega _{n}$ for $n< m$, we can define *ζ* on ϒ by setting $\zeta =\zeta _{n}$ a.e.By the monotone convergence theorem, we have

$$\begin{aligned} \lim_{n\rightarrow +\infty } \int \bigl(\zeta _{n}( \upsilon ) \bigr)^{q}\,d\mu ^{\blacktriangle }(\upsilon ,t_{\alpha }) &= \int \lim_{n\rightarrow +\infty } \bigl(\zeta _{n}( \upsilon ) \bigr)^{q}\,d\mu ^{\blacktriangle }(\upsilon ,t_{\alpha }) \\ &= \int \bigl(\zeta (\upsilon ) \bigr)^{q}\,d\mu ^{\blacktriangle }( \upsilon ,t_{ \alpha }) \\ &=N_{q}^{\blacktriangle }(\zeta ,t_{\alpha }) \\ &\geq N^{\blacktriangle } \bigl(\phi ^{\prime },t_{\alpha } \bigr) \\ &> 0. \end{aligned}$$

Then $\zeta \in (L^{+})^{q}$. Moreover, if $\xi \in (L^{+})^{p}$ and $\xi \chi _{\omega _{n}}\longrightarrow \xi $, then, by the fundamental convergence theorem, we have

$$\begin{aligned} \int \phi ^{\prime }(\xi )\,d\mu ^{\blacktriangle }(\upsilon ,t_{\alpha }) &= \int \phi ^{\prime } \Bigl(\lim_{n\rightarrow +\infty }\xi \chi _{\omega _{n}} \Bigr)\,d\mu ^{ \blacktriangle }(\upsilon ,t_{\alpha }) \\ &= \int \lim_{n\rightarrow +\infty } \bigl(\phi ^{ \prime }(\xi \chi _{\omega _{n}}) \bigr)\,d\mu ^{\blacktriangle }(\upsilon ,t_{ \alpha }) \\ &=\lim_{n\rightarrow +\infty } \int \phi ^{ \prime }(\xi \chi _{\omega _{n}})\,d\mu ^{\blacktriangle }(\upsilon ,t_{ \alpha }) \\ &=\lim_{n\rightarrow +\infty } \int \xi \chi _{ \omega _{n}}\zeta \,d\mu ^{\blacktriangle }(\upsilon ,t_{\alpha }) \\ &=\lim_{n\rightarrow +\infty } \int _{\omega _{n}} \xi \zeta \,d\mu ^{\blacktriangle }(\upsilon ,t_{\alpha }) \\ &= \int _{\Upsilon }\xi \zeta \,d\mu ^{\blacktriangle }(\upsilon ,t_{ \alpha }). \end{aligned}$$

Finally, suppose that $\mu ^{\blacktriangle }$ is arbitrary and $p>1$ is such that $q<+\infty $. As above, for each *σ*-bounded set $\omega \subset \Upsilon $, there is a unique $\zeta _{\omega }\in (L^{+})^{q}(\omega )$ such that $\int \phi ^{\prime }(\xi )\,d\mu ^{\blacktriangle }(\upsilon ,t_{\alpha })= \int (\xi \zeta _{\omega })(\upsilon )\,d\mu ^{\blacktriangle }(\upsilon ,t_{ \alpha })$ for all $\xi \in (L^{+})^{p}(\omega )$ and $N_{q}^{\blacktriangle }(\zeta _{\omega },t_{\alpha })\geq N^{ \blacktriangle }(\phi ^{\prime },t_{\alpha })$.If *κ* is *σ*-bounded and $\omega \subset \kappa $, then $\zeta _{\kappa }=\zeta _{\omega }$ a.e. on *ω* and $N_{q}^{\blacktriangle }(\zeta _{\kappa },t_{\alpha })\leq N_{q}^{ \blacktriangle }(\zeta _{\omega },t_{\alpha })$. Let $m= \inf \lbrace N_{q}^{\blacktriangle }(\zeta _{\omega },t_{\alpha }), \omega \subseteq \Upsilon \mbox{ be }\sigma \mbox{-bounded}\rbrace $, then $m\nleq N^{\blacktriangle }(\phi ^{\prime },t_{\alpha })$. Choose a sequence $\lbrace \omega _{n}\rbrace $ such that $N_{q}^{\blacktriangle }(\zeta _{\omega _{n}},t_{\alpha }) \longrightarrow m$ and set $\kappa =\bigcup_{n=1}^{+\infty } \omega _{n}$. Then *κ* is *σ*-bounded and $N_{q}^{\blacktriangle }(\zeta _{\kappa },t_{\alpha })\leq N_{q}^{ \blacktriangle }(\zeta _{\omega _{n}},t_{\alpha })$ for all $n\in \mathbb{N}$, whence $N_{q}^{\blacktriangle }(\zeta _{\kappa },t_{\alpha })=m$. Now, if *A* is a *σ*-bounded set containing *κ*, we have

$$\begin{aligned} N_{q}^{\blacktriangle }(\zeta _{A},t_{\alpha }) &= \int \bigl(\zeta _{A}( \upsilon ) \bigr)^{q}\,d\mu ^{\blacktriangle }(\upsilon ,t_{\alpha }) \\ &= \int \bigl( \bigl(\zeta _{\kappa }(\upsilon ) \bigr)^{q}+ \bigl(\zeta _{A-\kappa }( \upsilon ) \bigr)^{q} \bigr)\,d\mu ^{\blacktriangle }(\upsilon ,t_{\alpha }) \\ &= \biggl( \int \bigl(\zeta _{\kappa }(\upsilon ) \bigr)^{q} \biggr)\ast \biggl( \int \bigl( \zeta _{A-\kappa }(\upsilon ) \bigr)^{q} \biggr)\,d\mu ^{\blacktriangle }( \upsilon ,t_{\alpha }) \\ &\geq m=N_{q}^{\blacktriangle }(\zeta _{\kappa },t_{\alpha }) \\ &= \int \bigl(\zeta _{\kappa }(\upsilon ) \bigr)^{q}\,d\mu ^{\blacktriangle }( \upsilon ,t_{\alpha }). \end{aligned}$$

Thus $\int (\zeta _{A-\kappa })(\upsilon )\,d\mu ^{\blacktriangle }(\upsilon ,t_{ \alpha })=1$ and $\zeta _{A-\kappa }=0$ a.e. or $\zeta _{A}=\zeta _{\kappa }$ a.e. (here we used the fact that ${q<+\infty }$). But if $\xi \in (L^{+})^{p}$, then $A=\kappa \cup \lbrace \upsilon : \xi (\upsilon ) \neq0 \rbrace $ is *σ*-bounded and $\int \phi ^{\prime }(\xi )\,d\mu ^{\blacktriangle }(\upsilon ,t_{\alpha })= \int (\xi \zeta _{A})(\upsilon )\,d\mu ^{\blacktriangle }(\upsilon ,t_{ \alpha })=\int (\xi \zeta _{\kappa })(\upsilon )\,d\mu ^{\blacktriangle }( \upsilon ,t_{\alpha })$. Thus $\zeta =\zeta _{\kappa }$ and the proof is complete. □

## Conclusions

In this paper, we applied the theory of ∗-fuzzy measure to study a mathematical model related the outbreak of COVID-19. Next, we considered some properties of ∗-fuzzy measure and fuzzy integration, in particular we have shown the relationship between two ∗-fuzzy measures defined on the same ∗-fuzzy measure space and we have proved the fuzzy Lebesque–Radon–Nikodym theorem.

## Data Availability

Not applicable.
